# Supplementary material for: The Prognostic Value of the GNRI in Patients with Stomach Cancer Undergoing Surgery
Source: J Pers Med. 2023 Jan 13;13(1):155. doi: 10.3390/jpm13010155 (PMC9861269; doi:10.3390/jpm13010155)
Supplement: Supplementary file 1 [file jpm-13-00155-s001.zip › jpm-2100469-supplementary figures-main.pdf]

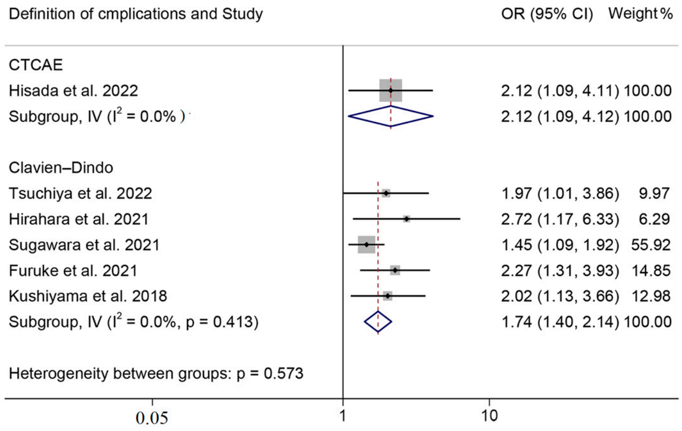

Figure S1. Subgroup analysis of postoperative complications based on the definition of complications. OR, odds ratio; CL, confidence interval [14,17,19-21,23].

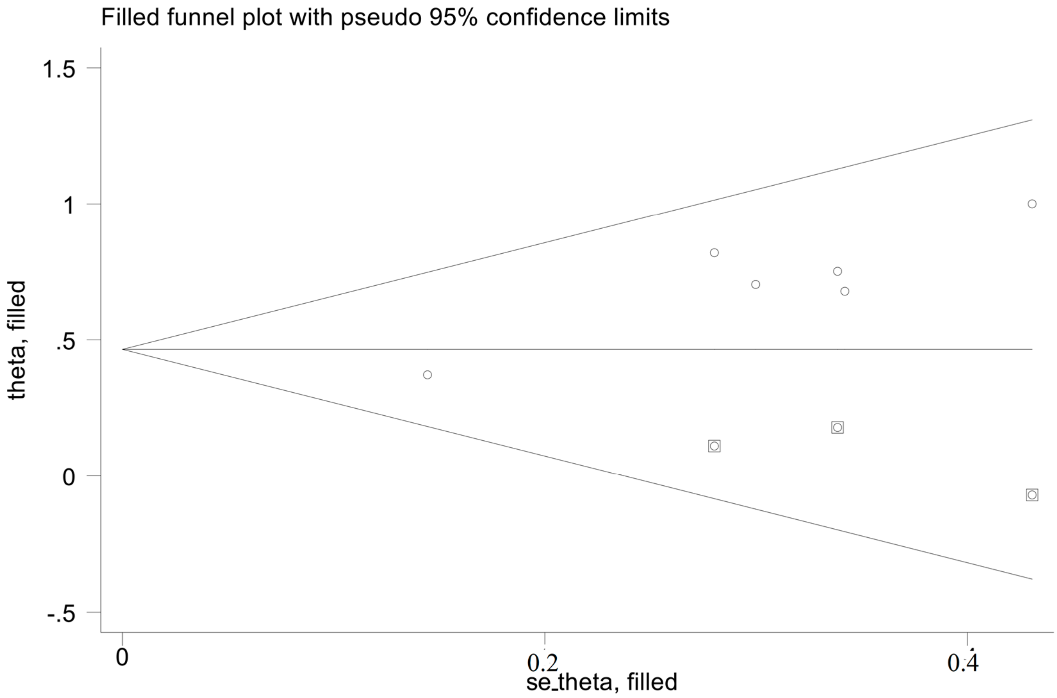

Figure S2. The picture of the trim and fill method. Theta, the effect estimate; Se_theta, the corresponding standard error; The circles represent the studies included in this meta-analysis; Boxes with circles represent additional studies of the trim and fill method.
